# SalivaDB—a comprehensive database for salivary biomarkers in humans

**DOI:** 10.1093/database/baad002

**Published:** 2023-02-07

**Authors:** Akanksha Arora, Dashleen Kaur, Sumeet Patiyal, Dilraj Kaur, Ritu Tomer, Gajendra P S Raghava

**Affiliations:** Department of Computational Biology, Indraprastha Institute of Information Technology, Okhla Phase 3, New Delhi 110020, India; Department of Computational Biology, Indraprastha Institute of Information Technology, Okhla Phase 3, New Delhi 110020, India; Department of Computational Biology, Indraprastha Institute of Information Technology, Okhla Phase 3, New Delhi 110020, India; Department of Computational Biology, Indraprastha Institute of Information Technology, Okhla Phase 3, New Delhi 110020, India; Department of Computational Biology, Indraprastha Institute of Information Technology, Okhla Phase 3, New Delhi 110020, India; Department of Computational Biology, Indraprastha Institute of Information Technology, Okhla Phase 3, New Delhi 110020, India

## Abstract

Saliva as a non-invasive diagnostic fluid has immense potential as a tool for early diagnosis and prognosis of patients. The information about salivary biomarkers is broadly scattered across various resources and research papers. It is important to bring together all the information on salivary biomarkers to a single platform. This will accelerate research and development in non-invasive diagnosis and prognosis of complex diseases. We collected widespread information on five types of salivary biomarkers—proteins, metabolites, microbes, micro-ribonucleic acid (miRNA) and genes found in humans. This information was collected from different resources that include PubMed, the Human Metabolome Database and SalivaTecDB. Our database SalivaDB contains a total of 15 821 entries for 201 different diseases and 48 disease categories. These entries can be classified into five categories based on the type of biomolecules; 6067, 3987, 2909, 2272 and 586 entries belong to proteins, metabolites, microbes, miRNAs and genes, respectively. The information maintained in this database includes analysis methods, associated diseases, biomarker type, regulation status, exosomal origin, fold change and sequence. The entries are linked to relevant biological databases to provide users with comprehensive information. We developed a web-based interface that provides a wide range of options like browse, keyword search and advanced search. In addition, a similarity search module has been integrated which allows users to perform a similarity search using Basic Local Alignment Search Tool and Smith–Waterman algorithm against biomarker sequences in SalivaDB. We created a web-based database—SalivaDB, which provides information about salivary biomarkers found in humans. A wide range of web-based facilities have been integrated to provide services to the scientific community. https://webs.iiitd.edu.in/raghava/salivadb/

## Introduction

People all over the world go through a variety of invasive and painful procedures for the diagnosis of all types of ailments. Procedures like frequent drawing of blood, biopsies and lumbar puncture contribute to the already stressful experience for patients (1, 2). There is a grave requirement to find non-invasive, cheap, precise and time-saving diagnostic methods. Saliva is one of the vital biofluids which can serve as a non-invasive biomarker for detecting a number of diseases (3). Human saliva is a clear liquid with a pH ranging between 6 and 7, and it constitutes 99% water and 1% of inorganic and organic molecules (4). About 97% of the whole saliva volume is secreted by the major glands (sublingual, submandibular and parotid), whereas the remaining 3% is secreted by minor salivary glands (labial, palatal, buccal and lingual) (5, 6). Saliva has a number of functions including tasting, swallowing, digestion, lubrication and protection against pathogens like bacteria (7). The salivary glands are covered with capillaries that allow the exchange of blood molecules into acinar cells that generate saliva (8).

In recent years, it has been shown that saliva contains a variety of biomolecules that can be used as disease biomarkers (9, 10). A number of molecules (e.g. enzymes, antibodies, hormones and growth factors) found in the blood enter saliva using paracellular (extracellular ultrafiltration) and transcellular (active and passive transport) pathways (10). These molecules may act as the circulating biomarkers of disease and contain sufficient information to detect disease status in patients (11). Saliva-based biomarkers have several advantages over blood which is a well-known biofluid for the diagnosis of diseases. The collection of saliva is non-invasive, inexpensive, has a reduced risk of pathogen transmission and is easier to store (12, 13). Moreover, it eliminates the need for a highly trained professional for the collection of the sample and can also be self-collected. Several past studies have shown that salivary biomarkers are able to accurately detect several diseases (14, 15). For example, a saliva-based enzyme-linked immunosorbent assay (ELISA) can be used to screen patients for human immunodeficiency virus (HIV)-1 and HIV-2 (10). Another study on salivary biomarkers for the detection of breast cancer concludes that eight messenger ribonucleic acid (RNA) biomarkers combined with one protein biomarker together achieved an accuracy of 92% (97% specific and 83% sensitive) (16). Saliva as a diagnostic and prognostic tool can not only save millions of lives but also reduce health-care cost by millions of dollars.

In the past years, several attempts have been made to compile saliva-associated information. The major resources containing information about saliva are Human Salivary Proteome Wiki, SalivaTecDB, The Human Metabolome Database (HMDB) and CancerPDF (17–21). Each resource provides unique information, for example, CancerPDF provides cancer-associated peptides/proteins in different body fluids including saliva. However, none of the existing resources provide updated information on all five types of saliva-based disease biomarkers. In order to complement existing resources, we developed a database SalivaDB that contains updated information about all kinds of saliva-based biomarkers. SalivaDB provides detailed and manually curated information obtained from the published research articles and existing resources.

## Methods

### Data collection

To collect all the literature related to salivary biomarkers, different relevant keywords were used to search research articles on PubMed. The keyword searches included ‘Salivary Biomarker AND Protein’ for proteins, ‘Salivary Biomarker AND miRNA’ for miRNA and ‘Salivary Biomarker AND (“microbe OR microbiota OR microorganism”)’ for microbes from 2017 to 2022, whereas ‘Salivary Biomarker AND gene’ keywords were used for genes and ‘Salivary Biomarker AND metabolite’ for metabolites for all years until 3 February 2022. These keywords were searched in the ‘All Fields’ filter and yielded 1423, 187, 111, 1274 and 170 publications for proteins, microRNA (miRNA), microbes, genes and metabolites, respectively. In addition to reading the articles extracted from PubMed, we also went through the references of each article to find other relevant research papers and ensure the proper coverage of the literature related to salivary biomarkers. After filtering the review, non-English and irrelevant articles from these publications, a total of 478 publications were studied that contained proper information about salivary biomarkers. Apart from this, we also added the data from SalivaTecDB, which covers information on the research and studies on protein, miRNA and microbe salivary biomarkers published until 2017, and the HMDB containing data for salivary metabolites to create an all-encompassing updated database.

### Database architecture

**Figure 1.**
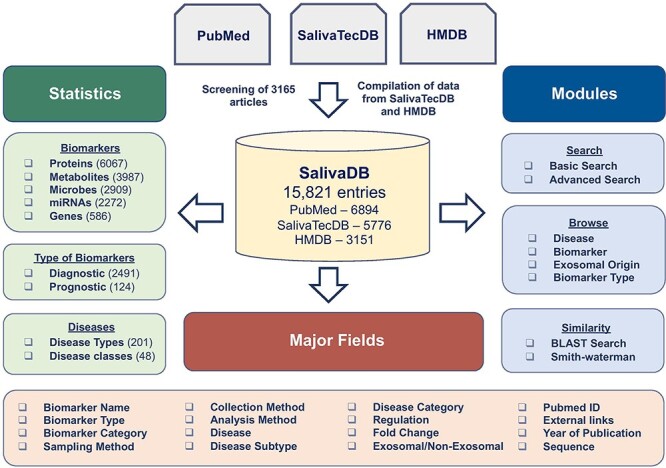
Architecture and content of SalivaDB.

SalivaDB was created using an Apache HTTP server (version 2.4.7), and MySQL (version 5.5.62) was used at the back end to manage the data. HTML5, PHP5, CSS3 and JAVA scripts were employed to create responsive front ends that are compatible with mobiles, tablets and Personal Computers. A combined interface was built using the PERL and PHP programming languages. The detailed architecture of SalivaDB is illustrated in Figure 1.

### Data content

This database contains experimentally validated information about genetic, proteomic, transcriptomic, metabolomic and microbial biomarkers for different diseases ranging from Alzheimer’s to cancer. The major fields in SalivaDB include (i) Biomarker name consists of the name of salivary biomarkers used in the literature; (ii) Biomarker type provides information about the type of biomarker, i.e. protein, metabolite, microbe, miRNA or gene; (iii) Sampling method constitutes the data about the number of control and diseased samples in the study; (iv) Collection method mentions the procedures used to collect saliva from the subjects; (v) Analysis method represents the methods used to analyse and experimentally validate the salivary biomarkers—e.g. ELISA, liquid chromatography with tandem mass spectrometry; (vi) Disease type represents the diseases or conditions in which a particular biomarker is present; (vii) Disease subtype reports the subtype of a particular disease if mentioned in the literature; (viii) Disease category represents the category that a specific disease or condition belongs to, e.g. neurological disorders, cancer, etc.; (ix) Fold change contains the data for how many folds a biomarker was reported to be upregulated or downregulated in the literature; (x) Up/Downregulated tells whether a biomarker was found to be up/downregulated for a condition or disease; (xi) External links contain links to external relevant sources of information like UniProt, PubChem, miRBase, National Center for Biotechnology Information Taxonomy and Gene databases; (xi) Sequences contain the sequences for proteins, genes, miRNA, microbes and Simplified Molecular Input Line Entry System for the metabolite biomarkers. Each entry is linked with the PubMed ID (PMID) from which the information of that particular biomarker was extracted (22–26).

## Results

### Data analysis

SalivaDB contains a total of 15 821 entries. Out of which, 6894 entries are manually curated from 478 articles on PubMed, 5776 entries have been taken from SalivaTecDB and 3151 entries have been extracted from HMDB. SalivaTecDB contains information about salivary biomarkers till the year 2017 for proteins, miRNA and microbes. We extracted the research papers for these three types of biomarkers from 2017 (1 January) to 2022 (3 February), as the exact date for SalivaTecDB data collection was not specified for the year 2017. We added the entries to our database after carefully analysing and selecting the entries for biomarkers that were only extracted from saliva and did not have a large number of empty fields. In addition to this, we also added information about metabolites and genes from all the research papers published till 3 February 2022.

**Figure 2.**
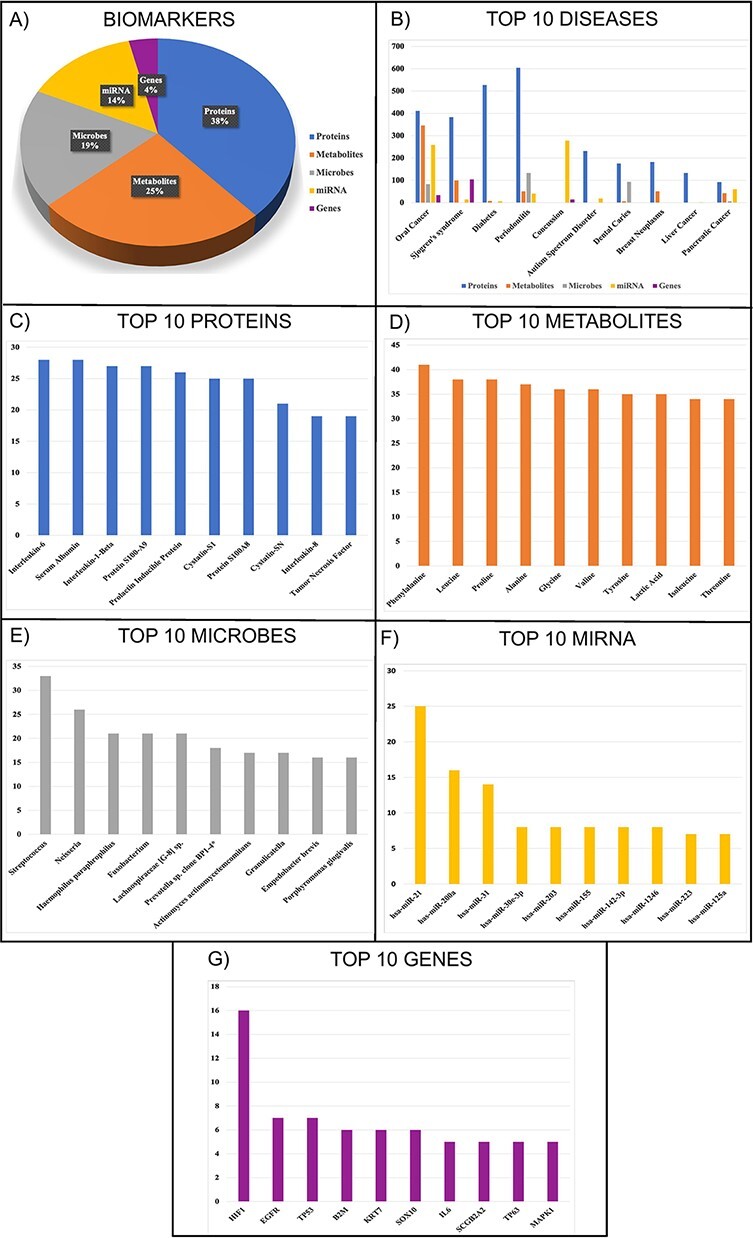
Statistics of (A) biomarkers in SalivaDB, (B) top 10 diseases, (C) top 10 proteins, (D) top 10 metabolites, (E) top 10 microbes, (F) top 10 miRNA and (G) top 10 genes in SalivaDB based on the number of entries.

Overall, the database contains 6067 (38%), 3987 (25%), 2909 (19%), 2272 (14%) and 586 (4%) entries for proteins, metabolites, microbes, miRNAs and genes, respectively, for 201 different diseases and 48 disease categories. The entries constitute of 7729 unique salivary biomarkers. It contains 2491 diagnostic, 124 prognostic and 159 both types of biomarkers. Moreover, SalivaDB provides information about 742 salivary biomarkers obtained from exosomes. Most salivary biomarkers have been reported for oral cancer, Sjogren’s syndrome, diabetes, periodontitis, concussion, etc. The percentage of biomarker types, top 10 diseases and top 10 biomarkers found in the SalivaDB based on the number of entries are shown in Figure 2. The top 20 biomarkers that had the highest number of entries and recurred in the literature are listed in Table 1, along with the related diseases.

**Table 1.** Top 20 salivary biomarkers in SalivaDB based on the number of entries

S. No.	Biomarker ID/biomarker name/type of biomarker/number of entries	Associated diseases or conditions
1	6140 (PubChem)/phenylalanine/metabolite/41	Oral cancer, pancreatic cancer, Sjogren’s syndrome, Yusho disease, Parkinson’s disease, thyroid cancer, lung cancer, breast cancer, periodontitis, oral leukoplakia (OLK), Alzheimer’s disease, dementia and Lewy body disease
2	6106 (PubChem)/leucine/metabolite/38	Oral cancer, Yusho disease, salivary gland tumour, thyroid cancer, lung cancer, breast cancer, periodontitis, OLK, Alzheimer’s disease, dementia and Lewy body disease
3	145 742 (PubChem)/proline/metabolite/38	Oral cancer, pain, Sjogren’s syndrome, Yusho disease, salivary gland tumour, head and neck cancer, chronic apical abscess, thyroid cancer, breast cancer, pancreatic cancer, periodontitis, OLK, Alzheimer’s disease, Lewy body disease, missing teeth and tooth decay
4	5950 (PubChem)/alanine/metabolite/37	Oral cancer, Yusho disease, salivary gland tumour, Parkinson’s disease, Sjogren’s syndrome, thyroid cancer, pancreatic cancer, breast cancer, OLK, Alzheimer’s disease, dementia, Lewy body disease and temporomandibular joint disorder
5	750 (PubChem)/glycine/metabolite/36	Oral cancer, Sjogren’s syndrome, Yusho disease, chronic apical abscess, Parkinson’s disease, thyroid cancer, breast cancer, pancreatic cancer, Alzheimer’s disease, dementia, Lewy body disease, periodontitis
6	6287 (PubChem)/valine/metabolite/36	Oral cancer, pain, Sjogren’s syndrome, Yusho disease, salivary gland tumour, breast cancer, pancreatic cancer, periodontitis, OLK, Alzheimer’s disease, dementia, Lewy body disease and missing teeth
7	6057 (PubChem)/tyrosine/metabolite/35	Oral cancer, breast cancer, Sjogren’s syndrome, Parkinson’s disease, lung cancer, pancreatic cancer, periodontitis, Alzheimer’s disease, dementia, Lewy body disease and missing teeth
8	107 689 (PubChem)/lactic acid/metabolite/35	Oral cancer, OLK, Alzheimer’s disease, dementia, Lewy body disease, temporomandibular joint disorder, missing teeth and periodontitis
9	6306 (PubChem)/isoleucine/metabolite/34	Oral cancer, breast cancer, Sjogren’s syndrome, Yusho disease, thyroid cancer, lung cancer, pancreatic cancer, periodontitis, OLK, Alzheimer’s disease, dementia, Lewy body disease and missing teeth
10	6288 (PubChem)/threonine/metabolite/34	Oral cancer, breast cancer, Sjogren’s syndrome, Yusho disease, salivary gland tumour, chronic apical abscess, thyroid cancer, pancreatic cancer, periodontitis, OLK, Alzheimer’s disease, dementia, Lewy body disease and missing teeth
11	1301 (Microbe)/*Streptococcus*/microbe/33	Dental caries, inflammatory bowel disease, lung cancer, polycystic ovary syndrome, epithelial precursor lesion and Parkinson’s disease
12	6262 (PubChem)/ornithine/metabolite/31	Oral cancer, Sjogren’s syndrome, Yusho disease, periodontitis, oral epithelial dysplasia, persistent oral mucosal lesions, chronic apical abscess, breast cancer, pancreatic cancer, Alzheimer’s disease, dementia and Lewy body disease
13	5962 (PubChem)/lysine/metabolite/29	Oral cancer, breast cancer, Sjogren’s syndrome, Yusho disease, chronic apical abscess, lung cancer, periodontitis, Alzheimer’s disease, dementia, Lewy body disease and missing teeth
14	P05231 (UniProt)/interleukin-6/protein/28	Oral cancer, periodontitis, systemic lupus erythematosus, Sjogren’s syndrome, rheumatoid arthritis, acute dental pain, obesity, gingivitis, temporomandibular joint osteoarthritis, physical frailty, bacterial infection, Huntington’s disease, liver transplant, Type 2 diabetes, systemic inflammation, dentofacial deformities, denture stomatitis and head and neck cancer
15	P02768 (UniProt)/serum albumin/protein/28	Oral cancer, oral lichen planus, fibromyalgia, periodontitis, Sjogren’s syndrome, alcohol and tobacco dependent, pancreatic cancer, acute physiological stress, Type 2 diabetes, dental caries, breast cancer and autism spectrum disorder
16	P01584 (UniProt)/interleukin-1-beta/protein/27	Oral cancer, periodontitis, stress, lung cancer, gingivitis, acute dental pain, physical frailty, liver transplant, dentofacial deformities, peri-implantitis, denture stomatitis and head and neck cancer
17	P06702 (UniProt)/protein S100-A9/protein/27	Oral cancer, Sjogren’s syndrome, periodontitis, liver cancer, antibody deficiencies, pregnancy, pancreatic cancer, acute physiological stress, Type 2 diabetes, dental caries, autism spectrum disorder, breast cancer, Type 1 diabetes and periodontitis
18	P12273 (UniProt)/prolactin inducible protein/protein/26	Oral cancer, multiple sclerosis, oral lichen planus, keratoconus, Sjogren’s syndrome, alcohol and tobacco dependent, antibody deficiencies, acute physiological stress, dental caries, breast cancer, autism spectrum disorder and periodontitis
19	5951 (PubChem)/serine/metabolite/26	Oral cancer, Sjogren’s syndrome, Yusho disease, salivary gland tumour, premalignant lesion, breast cancer, pancreatic cancer, periodontitis disease, Alzheimer’s disease, dementia and Lewy body disease
20	1060 (PubChem)/pyruvic acid/metabolite/26	Yusho disease, Alzheimer’s disease, dementia, Lewy body disease and salivary gland tumour

### Web interface

#### Browse

In SalivaDB, data can be browsed by (i) Biomarker Category—this module allows the user to choose the category of biomarkers which includes gene, protein, miRNA, metabolite and microbe; (ii) Biomarker Type—this module contains three options encompassing diagnostic, prognostic and both types of biomarkers; (iii) Disease Category—this allows user to select biomarkers from a wide range of disorders affecting humans; and (iv) Exosomal Origin—this module helps the user to choose the biomarkers that were derived from the extracellular vesicles—exosomes. These browse options result in a list of experimentally validated salivary biomarkers with expansive information, including their PMIDs, experiment details and more.

#### Search

In SalivaDB, basic and advanced search modules have been incorporated to make searching easier. The user can search for biomarkers by Biomarker Name, Biomarker Category, Biomarker Type, Disease Category, Disease Name, Type of Cancer and Exosomal Origin. In the basic search module, the output can be adjusted based on the search query. The advanced search feature allows users to simultaneously enter numerous queries using Boolean expressions (e.g. AND, NOT and OR).

#### Similarity search

To enable similarity-based search, the Basic Local Alignment Search Tool (BLAST) and Smith–Waterman algorithms have been implemented (27, 28). The user submits FASTA format protein/gene/miRNA sequence with default or specified settings to the BLAST module, and the server runs the BLAST search against stored data automatically. Similarly, the Smith–Waterman algorithm searches for proteins/genes/miRNA based on their similarity.

### Utility of SalivaDB

SalivaDB can act as an integrated platform where users can extract complete in-depth information about salivary biomarkers. For example, if a user wants to study and take out information about a miRNA called miR-1246, the user has to type miR-1246 in the search box and select the fields they want to be displayed on the screen. After clicking the search button on this page, the user will be redirected to the total number of entries for this particular biomarker. Users can click on Sal_ID to find out the detailed information for each entry. This page contains experiment details and various hyperlinks to PubMed and other databases like PubChem and UniProt, depending on the type of biomarker. The sequence of the biomarker is also available. Similarly, users can apply various operators (AND, OR and NOT) to narrow down their query in the advanced research tab. The browsing options like browse by disease type, biomarker category, exosomal origin and biomarker type are also available. The users can also search for the similarity of their query protein, miRNA and gene sequences in the similarity tab. The submodules of this tab include BLAST and the Smith–Waterman tool. It returns a list of biomarkers that are similar to the query, along with their scores.

### Comparison with other resources

There are several resources for salivary biomarkers, like Human Salivary Proteome Wiki and SalivaTecDB. However, there is no such database that provides comprehensive information about all types of salivary biomarkers. Human Salivary Proteome Wiki is a database maintained by the National Institutes of Health and contains only salivary proteins curated from UniProt, whereas SalivaTecDB contains manually curated data from research articles only till 2017 for proteins, miRNA and microbes. We created an updated database that covers all five types of biomarkers found in the saliva—genes, proteins, miRNA, metabolites and microbes which are lacking in the two above-mentioned databases. The complete comparison of SalivaDB with the existing resources is shown in Table 2. We also integrated the data from SalivaTecDB into our database to provide users with wide-ranging information about salivary biomarkers published in research articles up to date. SalivaDB aims to create a freely accessible, all-inclusive collection of all associated literature on biomarkers found in saliva to help discover and develop non-invasive methods for the diagnosis of diseases.

**Table 2.** Comparison of SalivaDB with the existing resources

	Salivary molecules					Biomarker type			
Database	Proteins	miRNA	Microbes	Genes	Metabolites	Diagnostic	Prognostic	Last update	Manual curation
SalivaDB	6067	2272	2909	586	3987	2491	124	2022	Yes
Human Salivary Proteome Wiki	3137							2022	No
SalivaTecDB	6521	192	3505			193		2017	Yes
HMDB					4756			2022	Yes
CancerPDF	170							2017	Yes

## Discussion

SalivaDB compiles information on five types of biomarkers found in saliva. All the data extracted from research papers are manually curated before being entered into the database. This database provides users with detailed data about salivary biomarkers collected from every published article related to this field. The data cover a wide variety of areas, including information about the patient cohort, methods used to sample and analyse the biomarkers, the regulation status of the biomarker and so on. This resource displays the data in tabular form, is freely available and is user-friendly, making it easy for people from all backgrounds.

### Applications

This database can be utilized for a variety of applications that will benefit the research communities. The following are a few of them:

(i) To the author’s knowledge, there is presently no other database that contains information on various types of signatures and biomarkers for saliva from such diverse domains as genomics, proteomics, transcriptomics, metabolomics and microbiomics.

(ii) This database has an easily comprehensible user interface that allows even users with little experience to use it conveniently.

(iii) The entries are linked with PubMed, UniProt, Gene, MiRBase, Taxonomy and PubChem, which make it easy for users to connect all the different platforms on a single platform—SalivaDB.

(iv) The advanced search tool enables the user to search complex queries on the database to narrow down and extract relevant data to their research.

(v) SalivaDB is especially beneficial for retrieving complete supporting information from the published literature to choose a specific biomarker for further research in the associated disease.

(vi) SalivaDB is helpful for people working in a wide variety of fields which include but are not restricted to disease diagnosis and prognosis, drug development, bioinformatics, study of omics, etc.

### Limitation

The data provided on SalivaDB are manually curated and thoroughly verified to reduce the risk of error. However, claiming complete accuracy would be unfair due to the possibility of human errors.

### Future direction

With researchers and scientists in search of non-invasive diagnostic methods for complex diseases, the literature on salivary biomarkers will grow. We will aim to update SalivaDB regularly with the availability of more research articles on this topic.

## Data Availability

All the datasets generated in this study are available at https://webs.iiitd.edu.in/raghava/salivadb/downloads.php.
